# A specific nanobody prevents amyloidogenesis of D76N β_2_-microglobulin *in vitro* and modifies its tissue distribution *in vivo*

**DOI:** 10.1038/srep46711

**Published:** 2017-04-21

**Authors:** Sara Raimondi, Riccardo Porcari, P. Patrizia Mangione, Guglielmo Verona, Julien Marcoux, Sofia Giorgetti, Graham W. Taylor, Stephan Ellmerich, Maurizio Ballico, Stefano Zanini, Els Pardon, Raya Al-Shawi, J. Paul Simons, Alessandra Corazza, Federico Fogolari, Manuela Leri, Massimo Stefani, Monica Bucciantini, Julian D. Gillmore, Philip N. Hawkins, Maurizia Valli, Monica Stoppini, Carol V. Robinson, Jan Steyaert, Gennaro Esposito, Vittorio Bellotti

**Affiliations:** 1 Department of Molecular Medicine, Institute of Biochemistry, University of Pavia, Via Taramelli 3b, 27100 Pavia, Italy; 2 Wolfson Drug Discovery Unit, Centre for Amyloidosis and Acute Phase Proteins, Division of Medicine, University College London, London NW3 2PF, UK; 3 Department of Chemistry, University of Oxford, Oxford OX1 3TA, UK; 4 Science and Math Division, New York University at Abu Dhabi, Abu Dhabi, UAE; 5 Structural Biology Research Centre, VIB, Pleinlaan 2, 1050, Brussel, Belgium; 6 Structural Biology Brussels, Vrije Universiteit Brussel, Pleinlaan 2, 1050, Brussel, Belgium; 7 Centre for Biomedical Science, Division of Medicine, University College London, London NW3 2PF, UK; 8 Department of Medical and Biological Sciences (DSMB), University of Udine, Piazzale Kolbe 4, 33100 Udine, Italy; 9 Istituto Nazionale Biostrutture e Biosistemi, Viale Medaglie d’Oro 305, 00136 Roma, Italy; 10 Department of Mathematics, Computer Science and Physics, University of Udine, Piazzale Kolbe 4, 33100 Udine, Italy; 11 Department of Biomedical, Experimental and Clinical Sciences ‘Mario Serio’, University of Florence, Viale Morgagni 50, 50134 Florence, Italy; 12 Research Centre for Molecular Basis of Neurodegeneration, 50134 Florence, Italy; 13 National Amyloidosis Centre, University College London, London NW3 2PF, UK

## Abstract

Systemic amyloidosis is caused by misfolding and aggregation of globular proteins *in vivo* for which effective treatments are urgently needed. Inhibition of protein self-aggregation represents an attractive therapeutic strategy. Studies on the amyloidogenic variant of β_2_-microglobulin, D76N, causing hereditary systemic amyloidosis, have become particularly relevant since fibrils are formed *in vitro* in physiologically relevant conditions. Here we compare the potency of two previously described inhibitors of wild type β_2_-microglobulin fibrillogenesis, doxycycline and single domain antibodies (nanobodies). The β_2_-microglobulin -binding nanobody, Nb24, more potently inhibits D76N β_2_-microglobulin fibrillogenesis than doxycycline with complete abrogation of fibril formation. In β_2_-microglobulin knock out mice, the D76N β_2_-microglobulin/ Nb24 pre-formed complex, is cleared from the circulation at the same rate as the uncomplexed protein; however, the analysis of tissue distribution reveals that the interaction with the antibody reduces the concentration of the variant protein in the heart but does not modify the tissue distribution of wild type β_2_-microglobulin. These findings strongly support the potential therapeutic use of this antibody in the treatment of systemic amyloidosis.

β_2_-microglobulin (β_2_m) causes a iatrogenic form of systemic amyloidosis when associated to long term haemodialysis^1^ and is associated with a familial form of the disease in the presence of the D76N mutation, characterized by progressive bowel disfunction and extensive amyloid deposits in the spleen, liver, heart, salivary glands and nerves^2^.

The mechanism of amyloid conversion of wild type β_2_m has been very extensively studied in the last two decades, however much of this work was performed under non-physiological conditions making it difficult to relate the findings to the pathological processes which occur *in vivo*. The possibility to reproduce *in vitro* the fibrillogenesis of β_2_m was exploited toward the identification and characterization of putative inhibitors suitable for drug development. To the best of our knowledge, characterization of inhibitors of β_2_m aggregation regarded only the full-length wild type β_2_m^3^,^4^ and its truncated form lacking the first six residues, ΔN6β_2_m, under the specific conditions necessary for fibrillogenesis *in vitro*. Indeed, full-length wild type β_2_m cannot be converted into amyloid fibrils in physiological conditions and therefore we cannot be completely confident that the effect *in vitro* may be reproduced *in vivo*. Methods compatible with the physiological environment are highly desirable for drug discovery and when we reported the first observed genetic variant of β_2_m^2^, we also found that the mutation confers to the protein a very high propensity to make amyloid fibrils in a physiologically relevant buffer^5^. Extensive investigation of the mechanism of fibrillogenesis of this genetic variant has revealed that biomechanical forces, compatible with those present *in vivo*^6^ can drive the amyloid conversion of this globular protein. We have therefore tested the efficacy of previously characterized inhibitors of the fibrillary conversion of wild type β_2_m^3^,^7^ on the fibrillogenesis of the natural D76N β_2_m variant causing hereditary systemic amyloidosis and found that complete abrogation of amyloid conversion can be achieved only by a specific monoclonal nanobody raised against the full-length wild type β_2_m^7^,^8^.

## Results

### Inhibition of β_2_m fibrillogenesis

Fibrillogenesis of D76N β_2_m was carried out in physiologically relevant conditions and fluid agitation^5^ in the presence and in the absence of ligands previously shown to inhibit wild type β_2_ fibrillogeneisis^3^,^7^,^8^. In particular, we tested the effect of doxycycline^3^ and nanobodies^7^,^8^ which were shown to be good inhibitors of wild type β_2_m fibril formation using procedures based either on the addition of 20% trifluoroethanol (TFE)^3^ or on low pH (pH 5)^7^.

**Figure 1:**
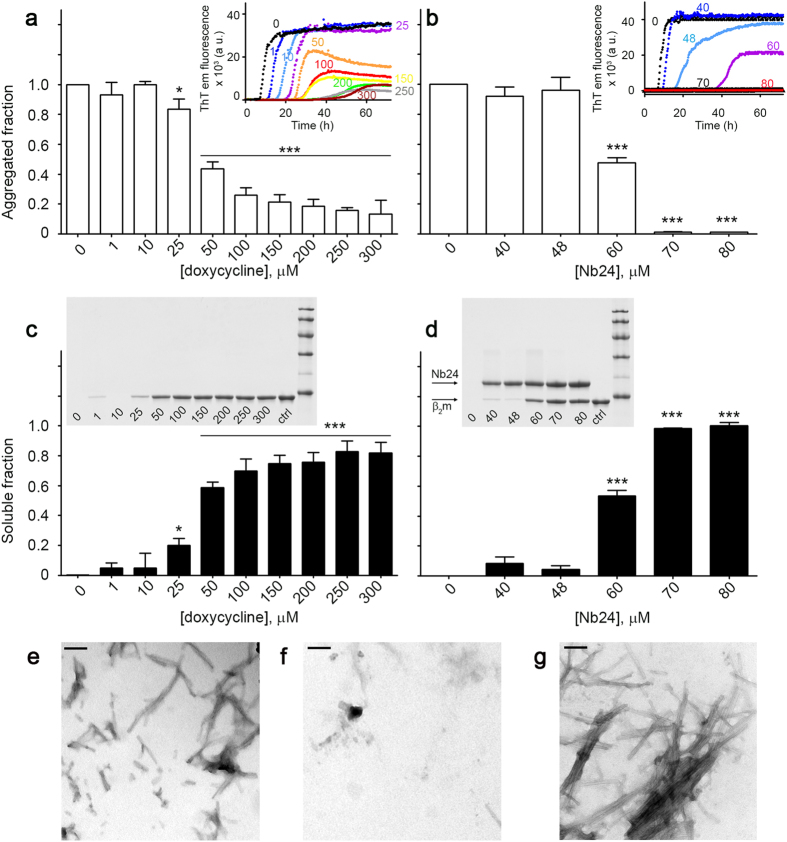
Inhibition of D76N β2m fibrillogenesis. (**a**) D76N β_2_m fibrillogenesis monitored by ThT emission fluorescence in the absence and in the presence of increasing concentrations of doxycycline or (**b**) Nb24 nanobody. Data were normalized to the thioflavin T signal plateau at ~72 h after the initiation of each reaction in the samples without any ligand. Means ± SD of three replicates are shown. *Insets*, representative sets of ThT emission fluorescence curves with the corresponding ligand concentration (μM). (**c,d**) Analysis of the soluble fraction remaining in the supernatant after 72 h in the absence and in the presence of doxycycline or Nb24 respectively. After centrifugation, supernatants were analyzed by SDS 15% homogenous PAGE; intensities of electrophoretic bands corresponding to monomeric β_2_m were quantified and normalized with the intensity of the band of the protein before aggregation. Means ± SD of three replicates are shown. *T*-test analysis: *P < 0.05; ***P < 0.001 *versus* sample containing D76N β_2_m only. *Insets*, SDS-PAGE are shown for each ligand with their corresponding concentrations. Protein before aggregation (*ctrl*) and marker proteins (14.4, 20.1, 30.0, 45.0, 66.0, and 97.0 kDa) are included. (**e–g**) Negatively stained transmission electron microscopy of the pellet harvested at the end of the fibrillogenesis (~72 h) in the presence of 300 μM doxycycline (**e**), 80 μM of Nb24 (**f**) or in the absence of any ligand (**g**) respectively (*scale bar*, 100 nm).

First of all, three nanobodies, Nb23, Nb24 and Nb30, previously selected for their capacity to inhibit the fibrillar conversion of the full-length wild type, truncated ΔN6 β_2_m and the non-natural variant P32G β_2_m^8^, were screened to determine whether they retained inhibitory activity against D76N variant β_2_m fibril formation. After 72 h of aggregation in physiological relevant conditions, Nb24 was found to be the only antibody able to inhibit the variant fibrillogenesis monitored with the thioflavin T (ThT) assay^9^ (Supplementary Fig. S1). This result prompted us to compare the effect of Nb24 and doxycycline on the fibrillogenesis of D76N β_2_m using a combination of ThT assay and quantification of the soluble fraction. ThT emission fluorescence decreased with increasing concentrations of doxycycline (Fig. 1a) showing that the drug inhibited amyloid formation by D76N β_2_m but did not completely abrogate its aggregation even at 300 μM, the highest concentration used (Fig. 1a). In contrast, 70 μM Nb24 inhibited amyloid formation completely (Fig. 1b). Assessment of soluble protein remaining after the end of the reaction (72 h) with either doxycycline or Nb24 was carried out using centrifugation followed by SDS-PAGE of the supernatants. Quantitative analysis of the SDS-PAGE bands corresponding to the monomeric β_2_m confirmed that Nb24 was a more potent inhibitor of D76N β_2_m fibrillogenesis (Fig. 1c,d). Negative stain transmission electron microscopy (TEM) of the pellet, harvested after centrifugation, showed that short fibrils could be still observed in the presence of 300 μM doxycycline (Fig. 1e), the highest experimental concentration used whereas fibrils were not present in the presence of two fold molar excess (80 μM) of antibody (Fig. 1f) compared to the classical fibrillar material formed after incubation of D76N β_2_m alone (Fig. 1g).

### Characterization of the interaction of Nb24 and D76N β_2_m

Since D76N β_2_m displays the fastest kinetics of aggregation so far described for natural β_2_m species and Nb24 is the only inhibitor able to inhibit the formation of fibrils, we have further characterized the binding properties of Nb24 with this variant using Biacore surface plasmon resonance, native mass spectrometry and NMR.

### Biacore

Analysis of the sensorgrams of the interaction between increasing concentrations of Nb24 and D76N β_2_m (Supplementary Fig. S2) immobilized on the sensor chips allowed us to determine both (mean ± SD) k_*on*_ (1.04 ± 0.22 × 10^5^ M^−1^ s^−1^) and k_*off*_ (9 ± 0.001 × 10^−3^ s^−1^) values resulting in a K_*D*_ of 87 ± 0.33 × 10^−9^ M. The affinity is therefore slightly lower than that measured with wild type β_2_m, K_*D*_ = 58 × 10^−9^ M^7^, and is consistent with the mutation D76N located within the epitope recognized by Nb24^7^.

### Native mass spectrometry

**Figure 2:**
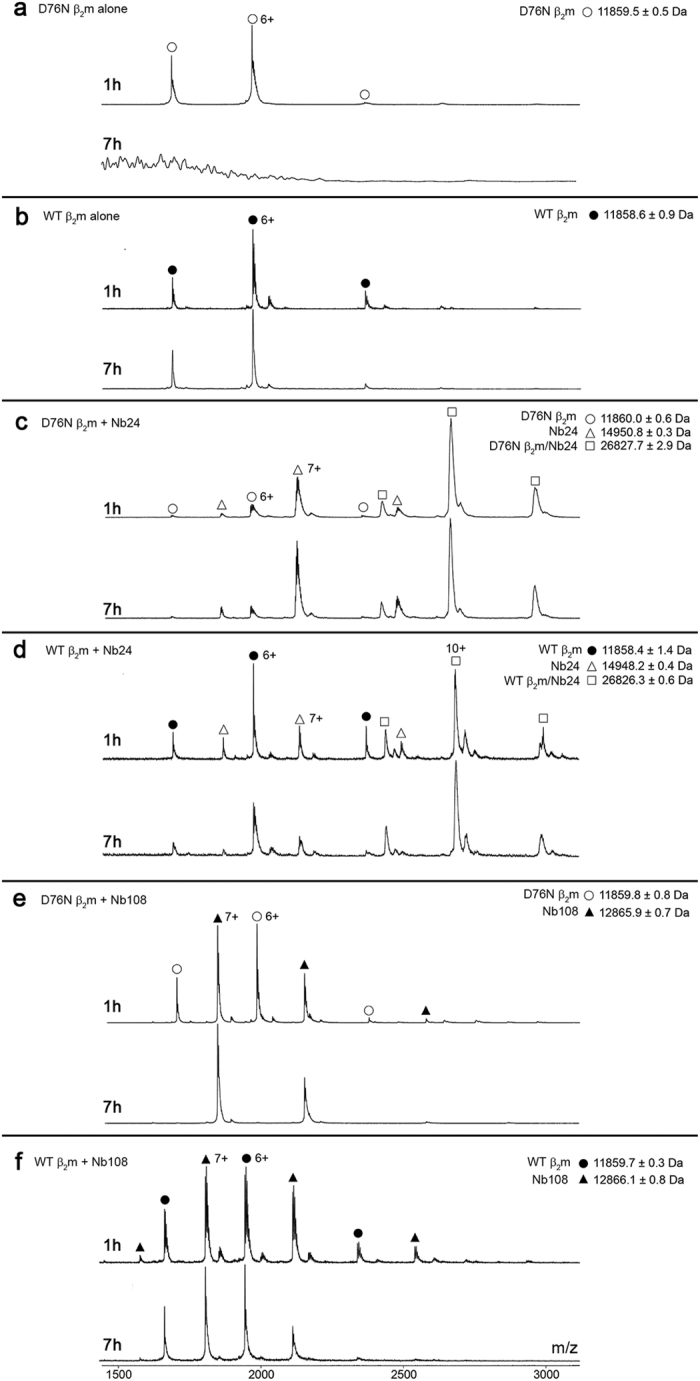
Native mass spectra analysis. All the samples were analyzed after 1 and 7 h incubation at 37 °C under stirring conditions. (**a,b**) D76N and wild type β_2_m alone; (**c,d**) D76N β_2_m or wild type β_2_m with twofold molar excess of Nb24; (**e,f**) D76N β_2_m or wild type β_2_m with twofold molar excess of unrelated Nb108. The variant precipitated over 7 h, at pH 7.4 with stirring, and the MS signal was lost (o, **a**); no soluble oligomers were observed in solution. In contrast, wild type β_2_m did not form fibrils under those conditions and there was no loss of MS signal (●, **b**). Both variant and wild type β_2_m formed a complex with Nb24 at 1 h as demonstrated by the MS signals labelled as □ (**c,d**). These signals were present at 7 h showing that the complex for both wild type and variant with Nb24 is stable. In contrast, there was no binding of Nb108 to either variant (**e**) or wild type β_2_m (**f**), as shown by the absence of ions for the complexed species. The loss of signal for the variant in the presence of Nb108 at 7 h is due to monomer precipitation as demonstrated above; only signals for the nanobody are present (**e**). Wild type β_2_m remained soluble (**f**). o (D76N β_2_m); ● (wild type β_2_m); Δ (Nb24); ▲ (Nb108); □ (β_2_m/Nb24 heterodimer).

The dynamics of β_2_m aggregation has been previously investigated by mass spectrometry^10^. However, due to the very slow kinetics of fibrillogenesis under physiologically relevant conditions *in vitro*, most of the studies have been performed in acidic conditions where the nucleation process is reasonably fast^11^. Since these conditions are not compatible with the functions of interactors designed for the inhibition of protein aggregation in physiological environment, we have monitored the incubation of both wild type and D76N variant β_2_m at pH 7.4 and 37 °C under stirring conditions. Untreated D76N β_2_m generated ions at m/z 2372.9 (5+), 1977.6 (6+) and 1695.2 (7+) corresponding to a MW of 11,859 ± 0.5 Da. These ions decreased in intensity over time and completely disappeared after 7 h, reflecting precipitation of the monomer (Fig. 2a). Although turbidity caused by fibrillar aggregates was macroscopically observed, no oligomers were visible by native MS (Fig. 2a), probably because the concentration of these transient species was too small compared to monomers and fibrils. As expected, the wild type did not form fibrils under those conditions; the sample remained clear after the overnight incubation and no decay was observed by native MS with ions at m/z 2372.8 (5+), 1977.5 (6+) and 1694.9 (7+) (MW 11,858 ± 0.9 Da) (Fig. 2b). To monitor the specificity of Nb24, both β_2_m isoforms were pre-incubated for 1 h with Nb24, or an unrelated nanobody, Nb108^7^ which was unable to inhibit the D76N variant fibrillogenesis (Supplementary Fig. S3), and then stirred under the same conditions.

The D76N variant formed a heterodimer with Nb24 (MW 26827.7 ± 2.9 Da) generating ions at m/z 2981.9 (9+), 2683.8 (10+) and 2439.9 (11+) and the complex remained soluble with no change in the relative ion intensities (Fig. 2c). The wild type protein also formed a stable complex with Nb24 (MW 26,826.3 ± 0.6 Da) generating ions at m/z 2981.8 (9+), 2683.6 (10+) and 2439.7 (11+) (Fig. 2d). When incubated with the unrelated Nb108 neither D76N nor the wild type interacted with the antibody as shown by the lack of ions related to the complexes. By following the intensity of the β_2_m monomers (5+ to 7+ charge states) incubated with Nb108, we could clearly observe loss of the amyloidogenic variant from solution after 7 h (Fig. 2e) whereas the wild type protein remained soluble, as expected under those conditions, with no change in the relative ion intensities (Fig. 2f).

### High resolution NMR of Nb24-β_2_m complexes

Based on the kinetic and thermodynamic constant values inferred from surface plasmon resonance (SPR) analysis, the expected NMR pattern should feature essentially quantitative complex formations upon titration of both β_2_m isoforms with Nb24, with an interaction regime of slow exchange on the NMR chemical shift time scale. Preliminary DOSY controls had ensured that this interaction occurred essentially with one-to-one stoichiometry (see Supplementary Information). The slow exchange regime should lead, at equilibrium, to the observation of separate resonance sets for the free and bound ^15^N-labeled β_2_m isoforms. No contribution to the spectra signal from unlabeled Nb24 was expected.

**Figure 3:**
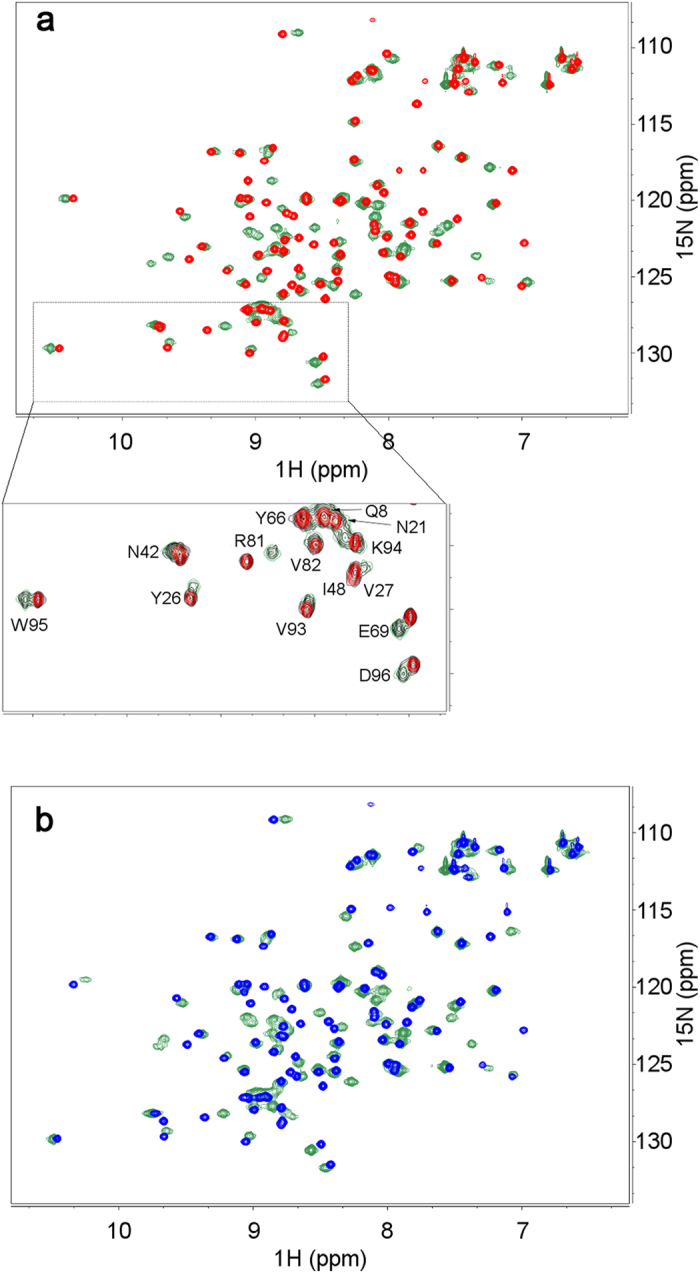
Effect of Nb24 binding on HSQC spectra of wild type and D76N β_2_m. ^15^N-^1^H HSQC map overlays of 70 μM wild type (**a**) and 75 μM D76N β_2_m (**b**) without and with unlabeled Nb24 nanobody (140 and 165 μM, respectively). In both panels the green contours are relative to the spectra with Nb24. All spectra were recorded at 600 MHz (^1^H frequency), 25 °C in 25 mM phosphate buffer (pH* 7.2). Nb24 forms a stable complex with both β_2_m species and the typical pattern observed along titration is depicted in the zoomed portion. The expanded region in panel (**a**) reports relevant contour overlay of Nb24 titration, namely at wild type β_2_m/Nb24 ratio of 1:0 (red), 1:0.7 (black), 1:2 (green), to illustrate the typical features of complexation induced perturbation. The single-letter code assignments refer to the ^15^N-^1^H correlation of the backbone amides, except for W95 that concerns side-chain (indole N^ε1^-H^ε1^). For the sake of clarity, labels are omitted for A15 and L23 that overlap Y66 and Q8 peaks, respectively.

Figure 3 shows overlays of the backbone and side-chain NH signals of ^15^N-^1^H HSQC spectra as obtained from wild type and D76N β_2_m solutions without and with the addition of a twofold Nb24 molar excess. The spectra confirmed the presence of stable complexes for both β_2_m isoforms in slow exchange with the free species. The expanded region in Fig. 3 illustrates the typical features observed throughout the titrations for wild type β_2_m, namely peaks exhibiting substantial, minor or essentially no chemical shift change upon addition of Nb24.

**Figure 4:**
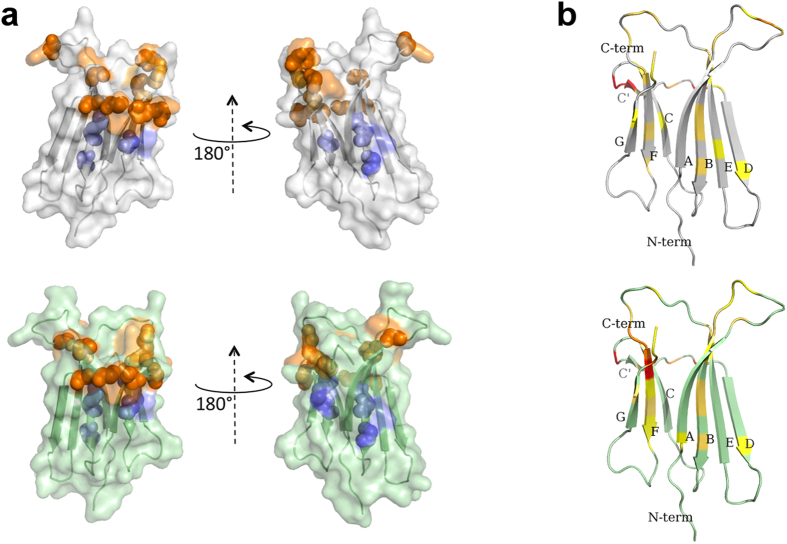
Epitope mapping of wild type and D76N β_2_m in their complexes with Nb24. (**a**) Structural map of the complexation induced shifts (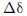) for the backbone amide ^15^N-^1^H NMR peak frequencies in wild type β_2_m (pale gray) and D76N β_2_m (pale green) upon interaction with Nb24 nanobody. For wild type β_2_m and D76N β_2_m, the average 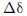 ± σ values of 0.08 ± 0.06 and 0.08 ± 0.06 ppm were obtained, respectively. NH locations with 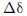 larger than one standard deviation from the average shift are indicated by spheres, with larger deviations by increasing color darkness. The two classes of chemical shift perturbations here presented indicate surface residues (orange-brown grading), and internal residues (cyan grading). For both β_2_m species, the effects of Nb24 binding are observed at the apical loops AB and EF and the C-terminal segment that represent a conformational epitope moiety. A further perturbation involves several consecutive residues of CD loop identifying a sequential epitope portion. Allosteric conformational effects involve internal residues at the end of strand B and, within strands C and F. (**b**) Elements of secondary structure in β_2_m and D76N β_2_m where residue locations are shown in yellow, orange and red when the corresponding backbone amide, upon Nb24 binding, exhibits a 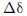 value larger than (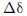
_av_ + σ), (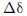
_av_ + 2σ) and (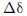
_av_ + 3σ), respectively.

These different effects on chemical shifts were accompanied by a general broadening of the signals with a complex pattern reflecting the extent of the inter- and intra-molecular dipolar interactions (Supplementary Fig. S4). In the absence of a precise structural model, it is very risky to attempt a rational quantitation of the individual signal intensity variations. On the contrary, changes in chemical shift of the signals, due to either average perturbation from ligand fast exchange or ligand complexation, may support a general, more reliable, qualitative inference for the interaction with Nb24. This approach leads to epitope mapping by chemical shift perturbation or complexation induced shifts^12^. Figure 4 illustrates the individual NH chemical shift deviations Δδ’s obtained from spectra of β_2_m species in the presence and in the absence of Nb24 and, classified according to the number of standard deviation (σ) from the average Δδ value. Hence displacements larger than σ, 2σ, 3σ or more indicate locations of the specific β_2_m species with progressively closer contacts with the interacting nanobody or/and larger deviations from the geometry of the isolated protein^12^.

Therefore the experimental data on the extent of chemical shift deviations for β_2_m and its D76N variant treated with Nb24 allowed us to reconstruct the epitopes involved.

Wild type β_2_m showed significant chemical shift perturbation at residues in the apical loops AB and EF as well as at the C-terminal segment, which together identified a possible conformational epitope (Fig. 4). In addition, a more remarkable perturbation involved several consecutive residues in loop C-D that might define a sequential epitope as another possible surface of contact with Nb24. Furthermore, large Δδs were also observed at the end of strand B, in strand C and strand F. These data are in part consistent with the crystallographic study carried out on the complex between Nb24 and P32G variant^8^.

Similarly, D76N β_2_m exhibited δ displacements at apical residues in loops AB and EF, at G strand and C-terminal that were consistent with a conformational epitope on the apex of the molecule. Again, a conspicuous perturbation occurred for C-D loop residues featuring a sequential epitope. Finally, other large displacements from the average deviation were observed at strand F (Fig. 4). The closely related patterns of Δδs observed for wild type and D76N β_2_m on binding to Nb24 (Supplementary Fig. S4) suggest a substantial analogy of the interaction mode of the nanobody with both species.

### Nb24 rescues partially unfolded D76N β_2_m

Consistently with evidence from SPR and mass spectrometry, the binding efficiency of Nb24 to both β_2_m isoforms was quite remarkable under the conditions used for the current NMR study and had an important effect on the stability of the amyloidogenic variant solutions. Indeed, we found that the conformational heterogeneity, which often characterizes preparations of D76N β_2_m could be abolished upon addition of Nb24. The onset of one or more additional conformers in freshly prepared or aged solutions of D76N β_2_m has been observed nearly always in our NMR determinations^5^ preluding to the shift of the natively folded conformation towards extensively unfolded species that eventually aggregate and precipitate. In our hands, with 0.05–0.3 mM D76N β_2_m solutions in phosphate buffer (20–50 mM) around neutrality (pH range 6.9–7.4), the first step of such a transformation pathway, i.e. the onset of the conformational heterogeneity, may develope over 5–10 days, a time lapse sensibly shortened at temperatures above 25–30 °C leading to the loss of the sample.

**Figure 5:**
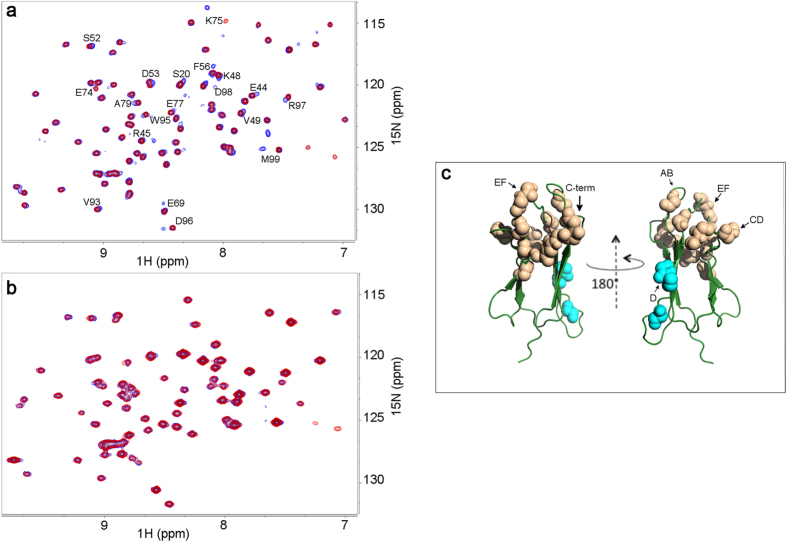
Nb24 rescues partially unfolded D76N β_2_m. (**a**) Overlay of ^15^N-^1^H HSQC spectral regions obtained from different D76N β_2_m samples, namely 57 μM with peak doubling because of partial unfolding onset (blue contours) and 75 μM with native conformation and no sign of heterogeneity (red contours). Both samples were prepared in 25 mM aqueous phosphate (pH* 7.2) and observed within a few days from preparation at 25 °C. The assignments for most of the doubled signals are reported. (**b**) The same D76N β_2_m solutions, as in panel A, were prepared with addition of Nb24 (see Methods), at analogous nanobody/protein concentration ratios, i.e. 114 μM/57 μM (blue contours) and 165 μM/75 μM (red contours), respectively. Peak doubling proved completely removed and the coincidence between the spectra of D76N β_2_m bound to Nb24 demonstrates that this nanobody rescues partial unfolding of the highly amyloidogenic β_2_m mutant. (**c**) Structural distribution of the D76N β_2_m residues involved in the early partial unfolding documented in panel A. The apical region (in pale brown) closely matches the epitope recognized by Nb24, whereas the D strand fragment involvement (in cyan) is an extension of the conformational heterogeneity from the preceding CD loop.

Figure 5a reports the overlay of two HSQC spectra obtained from D76N β_2_m samples at similar concentrations, respectively with and without traces of conformational heterogeneity. As illustrated, peak doublings were most clearly detectable for the C-terminal segment of the protein and the preceding residues of strand G. In addition, signals changed also in the adjacent N-terminal end of strand F and preceding loop residues, as well as in neighboring residues of strand C’, CD loop and C-terminal end of strand E (Fig. 5), i.e. a critical region that closely matches the epitope region recognized by Nb24. Further involvement of the initial fragment of strand D that in solution was largely lost^13^, appeared just a propagation of the conformational perturbation at the CD loop. By addition of Nb24, peak doublings were totally removed because the conformational equilibrium was quantitatively shifted by the binding species and the two HSQC maps became perfectly overlapping (Fig. 5b). The effect of Nb24 on the thermostability of D76N β_2_m was quite remarkable. HSQC spectra acquired in a range of increasing temperatures, from 25 °C to 57 °C, showed the loss of secondary and tertiary structures in the free monomeric protein compared to the protein in the complex which maintained its folded state (Supplementary Fig. S5).

### Stability, clearance and tissue distribution of the complex Nb24/β_2_m

The remarkable efficacy of Nb24 to protect the D76N variant from its amyloid conversion and its superiority in comparison to doxycycline prompted us to explore the stability in plasma and the clearance and tissue distribution of the β_2_m/Nb24 complex *in vivo* since these aspects are essential for the therapeutic exploitation for this type of nanobody.

To assess whether Nb24 was able to bind D76N β_2_m in plasma, the recombinant protein was incubated in human plasma (50 μg/ml) and, incubated at 37 °C in the presence or in the absence of twofold molar excess of Nb24. After centrifugation, supernatants were separated on a gel filtration column and fractions analyzed by western blotting following SDS-PAGE electrophoretic analysis. Control β_2_m eluted from the gel filtration at Ve ~ 15.1 ml and was separated from a higher molecular weight species (Ve ~ 13.6 ml) corresponding for the Nb24/β_2_m complex (Supplementary Fig. S6), showing that the complex had been formed in plasma.

**Figure 6:**
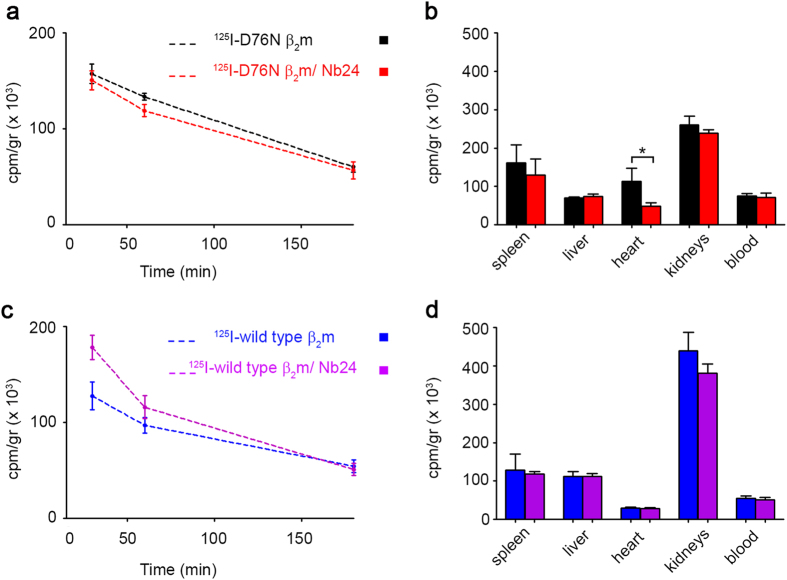
Clearance and tissue localization of ^125^I-β_2_m in β_2_m knock out mice. (**a**) Clearance of ^125^I-D76N β_2_m in groups of four mice (strain B6.129P2-B2m^tm1Unc^/J) receiving intravenously the protein either alone or in a 1:1 complex with Nb24. Clearance is not modified in the presence of the complex. (**b**) Localization of ^125^I-D76N β_2_m in tissues after 180 min. Mice treated with the complex have less radioactivity in the heart. (**c**) Clearance and (**d**) tissue localization of ^125^I-wild type β_2_m in groups of four mice (strain B6.129P2-B2m^tm1Unc^/J) receiving either the protein alone or in an equimolar complex with Nb24. Data expressed as cpm/gr represent mean ± SD of four mice per group. *P < 0.05 according to Kruskal-Wallis test followed by post-hoc Dunn’s test. ^125^I-D76N β_2_m alone, black; ^125^I-D76N β_2_m/Nb24, red; ^125^I-wild type β_2_m alone, blue; ^125^I-wild type β_2_m/Nb24, cyan.

Clearance and tissue distribution of ^125^I-D76N β_2_m *in vivo* was studied in groups of four knock-out mice receiving either the monomeric protein or the equimolar complex ^125^I-D76N β_2_m/Nb24. Gel filtration of plasma collected at the end of the study (180 min) from mice receiving the complex showed that the radioactive species eluted had the same elution volume of ^125^I-D76Nβ_2_m/Nb24 complex (Supplementary Fig. S7). The clearance of D76N β_2_m and the β_2_m/Nb24 complex were similar (Fig. 6a). When organs were counted at the end of the study (180 min), radioactivity was mostly found in the kidneys, which is the main pathway of clearance of circulating β_2_m, followed by spleen, heart and liver. Radioactivity in the heart of mice receiving ^125^I-D76N β_2_m alone was significantly higher than those given the pre-formed complex with Nb24 (Fig. 6b) in which total counts were reduced by approximately 60%. Comparative experiments carried out with ^125^I-wild type β_2_m showed that the Nb24 had appeared to slow β_2_m clearance; again, the kidneys were, as expected, the main tissue compartment for ^125^I-wild type β_2_m (Fig. 6c,d).

Histological examination of Congo red stained sections of the heart from mice receiving the protein alone or in complex with Nb24 did not show any evidence of aggregates as expected in the time frame of the experiment (Supplementary Fig. S8).

## Discussion

Despite remarkable progress achieved in the elucidation of the pathogenesis of systemic amyloidosis, the therapy of this very severe disease remains a challenging and unmet medical problem. Almost all the therapeutic strategies target the amyloidogenic protein in the attempt to minimize its expression^14^, or to stabilize its native folded state^15^ or even to impede the aggregation by chaperone-like synthetic or by biologic compounds^16^. For certain amyloidogenic proteins such as β_2_m the suppression of synthesis or stabilization by specific ligands is unfeasible and inhibitors of aggregation should be considered. The use of low molecular weight ligands such as doxycycline to slow and prevent wild type β_2_m fibrillogenesis has been demonstrated both *in vitro*^3^ and *in vivo*^17^. Nanobodies against β_2_m have been shown to be effective in blocking the amyloid transition of both wild type and the truncated β_2_-m isoform *in vitro*. We have evaluated the effect of one of these nanobodies, Nb24, on the amyloidogenesis of D76N β_2_m, a variant that causes a rare form of systemic amyloidosis which is not associated with hemodialysis, and for which no therapy is currently available^2^.

The affinity constant of D76N β_2_m for Nb24 was measured using surface plasmon reasonance and appears to be only slightly lower than that of the wild type protein^7^. Based on previous models of Nb24 in complex with P32G β_2_m and ΔN6 β_2_m^8^, the epitope contacting surface always involves the region around residue 76, i.e. the location of the variant mutation. Consistently, on complex formation, our NMR data show significant chemical shift changes in the backbone NHs of the same region including residue 76 NH for both β_2_m species (Supplementary Fig. S4). Therefore the lack of the negative charge of Asp76, replaced by Asn, may represent an important element responsible for the slightly reduced affinity of the variant species for the nanobody.

Our data clearly show that a therapeutic exploitation of doxycycline as therapeutic inhibitor of D76N β_2_m fibril formation is very unlikely since it cannot completely inhibit its amyloid transition *in vitro* under conditions of amyloidogenesis compatible with the biological environment. It is worth noting that even at high concentration of doxycycline, corresponding to the maximal inhibition of ThT signal, typical amyloid fibrils can be still observed. More promising and noticeably better results are achieved by using Nb24. In particular, in the presence of twofold molar excess of nanobody, a complete abrogation of amyloid formation can be achieved thus eliminating even a minimal residual formation of amyloid that is difficult to quantify but that is microscopically detectable at the end of the fibrillogenesis in the presence of the highest concentrations of doxycycline. The remarkable shift in the inhibition of D76N β_2_m fibrillogenesis from 60 to 70 μM Nb24 does reinforce the importance of minimizing the concentration of free β_2_m required for the formation of amyloid nuclei by maintaining a molar excess of antibody. The potent effect of fibrils in recruiting monomeric β_2_m may depend on the favorable thermodynamic state of fibrils *versus* both free D76N β_2_m^18^ and D76N β_2_m within the immuno-complex with Nb24 (Supplementary Fig. S9).

Furthermore, our NMR data suggest that Nb24 may prevent the conformational transitions leading to amyloidogenic species, and, furthermore, it may revert early conformational changes of the β_2_m variant occurring along the fibrillogenic pathway.

The complex D76N β_2_m/Nb24 is stable in human and mouse plasma and persists in circulation over the experimental time frame (Supplementary Fig. S7). The plasma clearance of the complex studied in β_2_m-knock-out mice is similar to that of free β_2_m, but tissue distributions reveals a difference in the amount of variant β_2_m that persists in the heart.

A comparative analysis of clearance and tissue distribution of wild type β_2_m in the absence and in the presence of the same antibody (Nb24) suggests that the effect on the cardiac localization of the variant may arise from its particular misfolding. We hypothesize that the variant β_2_m, once exposed to cardiac shear forces, might visit a partially folded state which is more prone to a strong interaction with the hydrophobic component of the extracellular matrix thus slowing down the protein efflux from the heart.

In conclusion Nb24 is more effective than doxycycline in inhibiting the amyloidogenesis of D76N β_2_m variant and the stability acquired by the amyloidogenic protein within the immune-complex reduces its permanence in the heart.

Our latest data suggest that Nb24 strongly prevents the induced cytotoxicity of D76N β_2_m aggregates described on the human SH-SY5Y cell line^19^ (Supplementary Fig. S10). In addition, previous studies showed that Nb24 cannot bind β_2_m within the MHC-I^7^ thus eliminating another potential adverse effect on the cells. Antibodies presenting the functional properties of Nb24 may be considered as candidate for an immunological therapy of familial amyloidosis caused by the D76N β_2_m variant.

## Material and Methods

### Protein preparation

Recombinant wild type and D76N β_2_m were expressed in transformed *E. coli* BL21DE3 strains and purified to homogeneity by sequential gel filtration and anion exchange chromatography^2^,^20^.

Uniformly ^15^N- labeled wild type or D76N β_2_m isoforms were also prepared using Spectra 9 minimal medium for NMR analysis.

Both wild type and D76N β_2_m were labelled with ^125^I using N-bromosuccinimide and sodium [^125^I] iodide (Perkin Elmer, Seer Green, UK) in PBS for 10–15 s and purified on a PD10 desalting column (Bio-Rad, Hemel Hempstead, UK)^21^; ^125^I-β_2_m was prepared at a specific activity of 16.0 MBq/mg. Approximately 1 μg (0.017 MBq) of ^125^I β_2_m was diluted into 100 μg of native recombinant β_2_m to give a final specific activity of ~0.17 MBq/mg for each mouse for *in vivo* clearance and tissue localization experiments.

The anti β_2_m nanobody, Nb24, originally selected from a phage display library constructed from a β_2_m immunized-camel, was expressed in *E. coli* as C-terminal His_6_-tagged protein using a pHEN6 cloning vector. The nanobody was purified to homogeneity by immobilized-metal affinity chromatography and gel filtration^7^.

### Inhibition of D76N β_2_m fibrillogenesis by nanobodies

A series of experiments were carried out with 20 μM β_2_m and twofold molar excess of Nb23, Nb24, Nb30 which all were previously shown to be good inhibitors of other β_2_m species^8^. Fibrillogenesis was performed in standard quartz cells stirred at 1,500 rpm and 37 °C in PBS, pH 7.4 containing 10 μM thioflavine T (ThT)^9^. ThT emission was monitored at 480 nm after excitation 445 nm, using a Perkin Elmer LS 55 spectrofluorimeter.

### Fibrillogenesis

Fibrillogenesis of recombinant D76N β_2_m was carried out in the absence and in the presence of doxycycline (0, 1, 10, 25, 50, 100, 150, 200, 250 and 300 μM) and nanobody Nb24 (40, 48, 60, 70 and 80 μM) respectively. Samples, 100 μl with 40 μM of recombinant protein in PBS pH 7.4 containing 10 μM ThT with or without ligands, were incubated at 37 °C in Costar 96-well black-wall plates sealed with clear sealing film and subjected to 900 rpm double orbital shaking. Bottom fluorescence was recorded at 15-min interval (FLUOstar Omega, BMG LABTECH). Since both doxycycline and Nb24 do not interfere with ThT spectrofluorimetric assay, amyloid formation was monitored by ThT emission^9^ at 480 nm after excitation at 445 nm in three or more replicates. The data were normalized to the signal plateau of the protein alone at 72 h. The protein remained soluble after the fibrillogenesis was analyzed by SDS homogenous 15% PAGE. The soluble fractions were separated by centrifugation at 10,600 × *g* for 20 min for the electrophoretic analysis; bands were quantified with Quantity One software (Biorad) and compared to the band of the protein before aggregation. The pellet harvested were resuspended with a minimal volume of water and analyzed by transmission electron microscopy (TEM) using a CM120 microscope at 80 keV after 2% w/v uranyl acetate staining. Nanobody Nb108, raised and selected against an unrelated antigen, was used at twofold molar excess as negative control in experiments of fibrillogenesis of D76N β_2_m using ThT fluorescence assay as described above.

### Biacore

The experiments were performed at 25°C on a BIAcoreX instrument (GE Healthcare, Piscataway, NJ, USA). D76N β_2_m was covalently immobilized on the dextran matrix sensorchip surface (CM5 chip) using a standard amine coupling protocol (Supplementary Information).

### Native mass spectrometry

β_2_m, Nb24 and Nb108 were resuspended in 200 mM ammonium acetate pH 7.4, at 40 μM concentration. Each β_2_m isoform was then incubated in the absence and in the presence of equimolar Nb24 or N108 solutions (final 2 ml volume), at 37 °C under stirring conditions (1,500 rpm, IKA magnetic stirrer). After different incubation times, 2 μl aliquots from the samples were loaded onto in-house pulled and coated silica needles^22^. The spectra were acquired on a LCT-TOF (Waters) modified for a better transmission of high m/z species. Parameters were as follows: capillary, sample cone and extraction cone voltages were set to 1.5 kV, 50 V and 5 V, respectively, to maintain non-covalent interactions. The pressure in the source was set to 5.5 mBar in order to increase the collisional cooling of the ions by reducing their internal energy.

### NMR

Samples were obtained by dissolving lyophilized protein in 25 mM phosphate buffer solution prepared in H_2_O/D_2_O 94/6 at pH* of 7.2 (pH* = uncorrected pHmeter reading). The proteins were either ^15^N-uniformly-labeled (wild type β_2_m and D76N β_2_m) or unlabeled (Nb24). Typically, concentration-adjusted Nb24 stock solutions (200–300 μM) were prepared and mixed with equal volumes of half-concentration-adjusted, ^15^N-labeled wild type or D76N β_2_m stock solutions (100–150 μM), to achieve with 2:1 Nb24/protein solutions (100/50–150/75). Protein blank solutions were prepared by dilution of the corresponding stock solutions and titrations were accomplished by adding increasing aliquots of Nb24/protein solutions to the appropriate blank solution.

NMR spectra were collected at 14.0 T, on the Bruker Avance III NMR facility of the Core Technology Platform at New York University Abu Dhabi. The spectrometer, equipped with cryoprobe and *z*-axis gradient unit, operated at 600.13 and 60.85 MHz to observe ^1^H and ^15^N, respectively. In addition to 1D control spectra, 2D ^15^N-^1^H HSQC^23^ were recorded over spectral widths of 40 ppm (^15^N, t_1_) and 15 ppm (^1^H, t_2_), and digitized over 192 and 2,048 points, respectively. For each t_1_ dimension point, 16 or 32 scans were accumulated and quadrature in the same dimension was accomplished by gradient-assisted coherence selection (echo-antiecho)^24^. Processing with t_1_ linear prediction, apodization and zero-filling prior to Fourier transformation led to 2K × 1K real spectra. Diffusion coefficients were determined by means of 2D ^1^H DSTEBPP (Double STimulated Echo BiPolar Pulse) experiments that besides correcting for eddy current artifacts also compensate the induced convection contributions^25^. The *z*-axis gradient strength was varied linearly from 2 to 98% of its maximum value (~60 G/cm) and matrices of 2,048 by 80 points were collected by accumulating 64 scans per gradient increment. The acquired data were processed using the Bruker software *Dynamics Center* (version 2.2) to extract the diffusion coefficients. Both curve fitting and inverse Laplace transform^26^ routines were employed, but only the results from the latter were retained because of the improved fitting reliability. Water suppression was achieved by appending to the DSTEBPP sequence a pair of WATERGATE^27^ elements in the excitation-sculpting mode^28^, as for 1D experiments, or using a flip-back pulse in the HSQC experiments^29^. All measurements were performed at 25 °C. The chemical shift perturbation analysis is given in term of combined chemical shift (δ) deviation, i.e. Δδ’s, calculated by cumulating both ^1^H and ^15^N Δδ values with proper scaling factors^30^.

### Stability of D76N β_2_m/Nb 24 complex in plasma

Human plasma equilibrated at 37 °C was sequentially filtered with 0.45 and 0.22 μm filters before the addition of either 50 μg/ml of D76N β_2_m alone or in complex with twofold molar excess of Nb24. After a 30 min incubation, aliquots of 50 μl from each sample were separated using a Superdex 75 on an ÄKTA Explorer apparatus (GE Healthcare). The column was equilibrated and eluted in PBS pH 7.4 at 0.3 ml/min. Fractions of 300 μl were collected and analyzed by SDS 8–18% Excel PAGE (GE Healthcare) and then blotted to PVDF membrane for identification with primary rabbit polyclonal anti β_2_m antibody (4.8 μg/ml Dako, Denmark) and secondary goat anti-rabbit IgG peroxidase conjugate (0.05 × 10^−3^ μg/ml. Western blotting was developed with SIGMA FAST 3,3′-diamino benzidin tablets (Sigma Aldrich).

### Clearance and tissue localization in β_2_m knock out mice

Groups of four mice (strain B6.129P2-B2m^tm1Unc^/J) received intravenously 100 μg of protein either alone (D76N or wild type β_2_m) or in a 1:1 complex with Nb24 respectively. Each dose contained trace amounts of the corresponding ^125^I-labeled β_2_m isoform. Plasma samples were collected at 30, 60 and 180 min for clearance studies. Gel filtration of plasma collected at 180 min was performed to assess the persistence of β_2_m in the complex (Supplementary Information). After 180 min, mice were killed and organs collected. Radioactivity was counted with a Perkin Elmer 2470 Automatic gamma counter. Before total ^125^I measurement all the organs were rinsed in PBS, blotted-dried and weighted. Data expressed as cpm/gr of blood or tissue represent mean ± SD of four mice per group. Animal studies were ethically reviewed and approved by the UCL Royal Free Campus Ethics and Welfare Committee and the UK Home Office, and complied fully with European Directive 86/609/EEC.

### Statistical analysis

Data analyses used GraphPad Prism version 5. Applied analyses are indicated in corresponding legends. Differences with P < 0.05 were considered statistically significant.

## Additional Information

**How to cite this article:** Raimondi, S. *et al*. A specific nanobody prevents amyloidogenesis of D76N β_2_-microglobulin *in vitro* and modifies its tissue distribution *in vivo. Sci. Rep.* **7**, 46711; doi: 10.1038/srep46711 (2017).

**Publisher's note:** Springer Nature remains neutral with regard to jurisdictional claims in published maps and institutional affiliations.

## Supplementary Material

Supplementary Information
