# Supplementary material for: A specific nanobody prevents amyloidogenesis of D76N β2-microglobulin in vitro and modifies its tissue distribution in vivo
Source: Sci Rep. 2017 Apr 21;7:46711. doi: 10.1038/srep46711 (PMC5399440; doi:10.1038/srep46711)
Supplement: Supplementary Information [file srep46711-s1.pdf]

SUPPLEMENTARY INFORMATION

A specific nanobody prevents amyloidogenesis of D76N β_2-microglobulin *in vitro* and modifies its tissue distribution *in vivo*

Sara Raimondi, Riccardo Porcari, P. Patrizia Mangione, Guglielmo Verona, Julien Marcoux, Sofia Giorgetti, Graham W. Taylor, Stephan Ellmerich, Maurizio Ballico, Stefano Zanini, Els Pardon, Raya Al-Shawi, J. Paul Simons, Alessandra Corazza, Federico Fogolari, Manuela Leri, Massimo Stefani, Monica Bucciantini, Julian D. Gillmore, Philip N. Hawkins, Maurizia Valli, Monica Stoppini, Carol V. Robinson, Jan Steyaert, Gennaro Esposito & Vittorio Bellotti

Supplementary methods

Biacore. D76N β_2m was covalently immobilized on the dextran matrix sensorchip surface (CM5 chip). The carboxyl groups on the surface of the flow cell were activated by injecting a 1:1 mixture of 100 mM N-ethyl-N'-(dimethylaminopropyl)-carbodiimide (EDC) and 100 mM N-hydroxysuccinimide (NHS); then a 20 µM D76N β_2m solution in 20 mM sodium acetate buffer pH 5.5 was injected over the surface where its level of immobilization was 8000 RU. Residual activated carboxy-methyl groups were blocked with 1 M ethanolamine, pH 8.5. Each step was performed with a 7-min injection at a 5 µl/min flow rate. Nb24 (4 min) was injected over the surface at the flow rate of 5 µl/min at different concentrations from 10 to 100 nM. The running buffer used was 10 mM HEPES pH 7.4 containing 150 mM NaCl, 5 mM EDTA, and 0.05% Tween 20. Sensorgrams from three sets of data for each of the Nb concentrations were collected. Association and dissociation kinetic rate constants (k_{on} and k_{off}) and the equilibrium dissociation constant K_D were calculated using the Langmuir equation with the BIAevaluation 3.0 software[1].

Thermostability of D76N β_2m with and without Nb24. Using the same D76N β_2m mother solution, two aliquots were prepared containing 45 µM of isolated protein and 44 µM β_2m with 93 µM Nb24 (D76N β_2m/ Nb24 ratio= 2:1). The pH=7.03 was maintained using 25 mM phosphate buffer. Both samples were submitted to NMR measurements at different temperatures ranging from 25 ºC up to 57 ºC for about 11 h of stepwise progressive heating (25, 31, 37, 42, 47, 52, 57 ºC). After 20 min of thermal equilibration at each temperature, 1D and 2D HSQC spectra were collected with an overall measurement time of 70 min.

Persistence of D76N β_2m/Nb24 complex *in vivo*. A 42 µM solution of D76N β_2m with and without equimolar quantity of Nb24 in the presence of ^{125}I-labelled D76N β_2m (2%) were

1/1 diluted in β$_2$m knock out mouse plasma. Sample of 100 µl were loaded into a Superdex 75 on an ÄKTA Explorer apparatus (GE Healthcare). The column was equilibrated and eluted in PBS pH 7.4 at 0.3 ml/min. Fractions of 300 µl were collected and analyzed by a Perkin Elmer 2470 Automatic gamma counter. Same procedure was applied to plasma collected at 180 min from β$_2$m knock out mice receiving ^{125}I-D76N β$_2$m/Nb24.

Cell culture and cell viability assay. The SH-SY5Y human neuroblastoma cells were cultured at 37 °C in complete medium (50% HAM, 50% DMEM, 10% fetal bovine serum, 3.0 mM glutamine, 100 units/mL penicillin and 100 µg/mL streptomycin), in a humidified, 5.0% CO_2 incubator. All reagents were from Sigma-Aldrich. The cytotoxicity of the different forms of D76N β$_2$m aggregates grown at different aggregation times was assessed by the 3-(4,5-dimethylthiazol-2-yl)-2,5-diphenyltetrazolium bromide (MTT) reduction inhibition assay based on the protocol described for the first time by Mosmann[2]. In all MTT-experiments, the cells were plated and incubated for 48 h at a density of 10,000 cells per well on 96-well plates in 100 µl culture medium. Then, the cells were treated for 24 h with (5 µM) D76N β$_2$m after 96 h of aggregation time in the absence or in the presence of Nb24 (1:0.5 or 1:2, β$_2$m: Nb24 molar ratio). After exposure to the aggregates, the cells were incubated for 2.0 h with 100 µl of DMEM without phenol red, containing 0.5 mg/ml MTT. Then, 100 µl of cell lysis buffer (20% SDS, 50% N,N-dimethylformamide, pH 4.7) was added to each well and the samples were incubated at 37 °C for 2 h to allow complete cell lysis. Absorbance values of blue formazan were determined at 595 nm with an automatic plate reader (Bio-Rad, Hercules, Cal). The final absorption values were calculated by averaging four independent measurements of each sample after subtraction of the average of the blank solution containing 100 µl of MTT solution and 100 µl of lysis buffer: 20% SDS, 50% N, N-dimethylformamide).

Supplementary figures

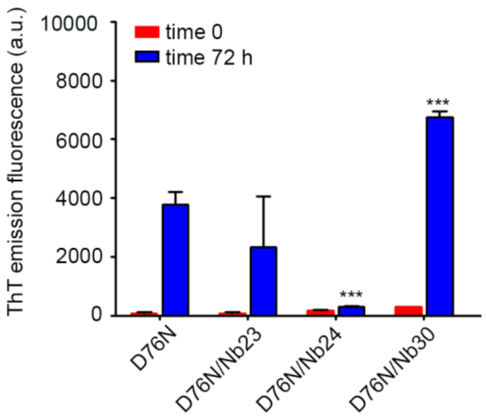

Supplementary Fig. S1. Inhibition of D76N β$_2$m fibrillogenesis by nanobodies. ThT emission fluorescence of samples containing 20µM D76N β$_2$m in the absence and in the presence of twofold molar excess of Nb23, Nb24, Nb30. Samples were incubated in PBS containing 10µM ThT, 37C under stirring condition; values before and at after 72 h are shown as mean ± S.D. *T*-test analysis: ****P* < 0.001 *versus* sample containing D76N β$_2$m only at 72 h. The ThT signal of the complex D76N β$_2$m with Nb30 after 72 h incubation was higher than the one with the protein alone suggesting that Nb30 may actually enhance the aggregation of the protein.

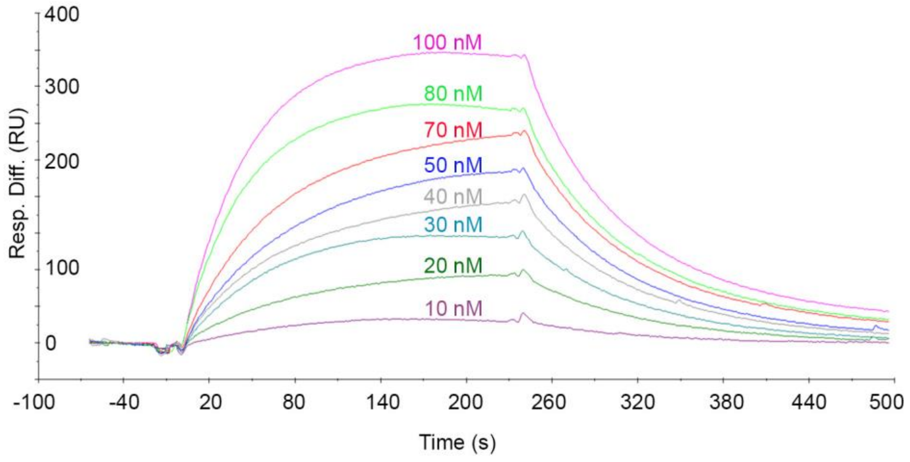

Supplementary Fig. S2. Binding of Nb24 with immobilized D76N β_2m. Overlay of sensorgrams recorded at increasing concentrations of Nb24. Contact time and dissociation time was kept at 4 min and 5 min. Each sensorgram was evaluated using the BIA evaluation 3.0 software provided with the system[1].

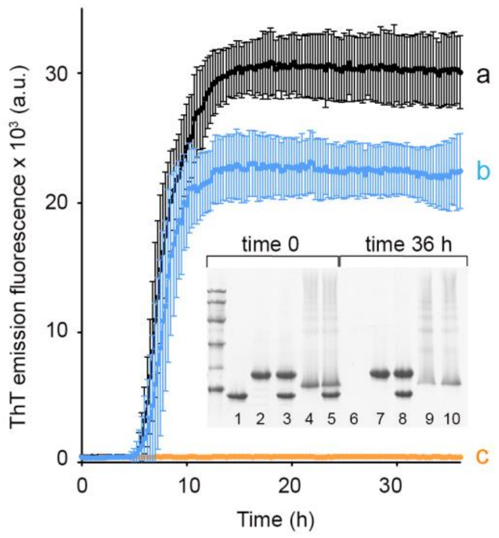

Supplementary Fig. S3. Unrelated nanobody, Nb108, does not inhibit β$_2$m fibrillogenesis. ThT fluorescence data of D76N β$_2$m (40 µM) in the absence (a) and in the presence of twofold molar excess of Nb108 (b) and Nb24 (c). Samples were incubated at 37 ºC in a microplate at 900 rpm double orbital shaking. Data plotted as mean ± SD of at least three replicates. Inset, SDS-15% PAGE under reducing conditions of samples analyzed at time 0 and after 36 h of incubation. D76N β$_2$m (1, 6), Nb24 (2, 7), D76N β$_2$m/ Nb24 (3, 8), Nb108 (4, 9), D76N β$_2$m/ Nb108 (5, 10); marker proteins (14.4, 20.1, 30.0, 45.0, 66.0 and 97.0 kDa) are included.

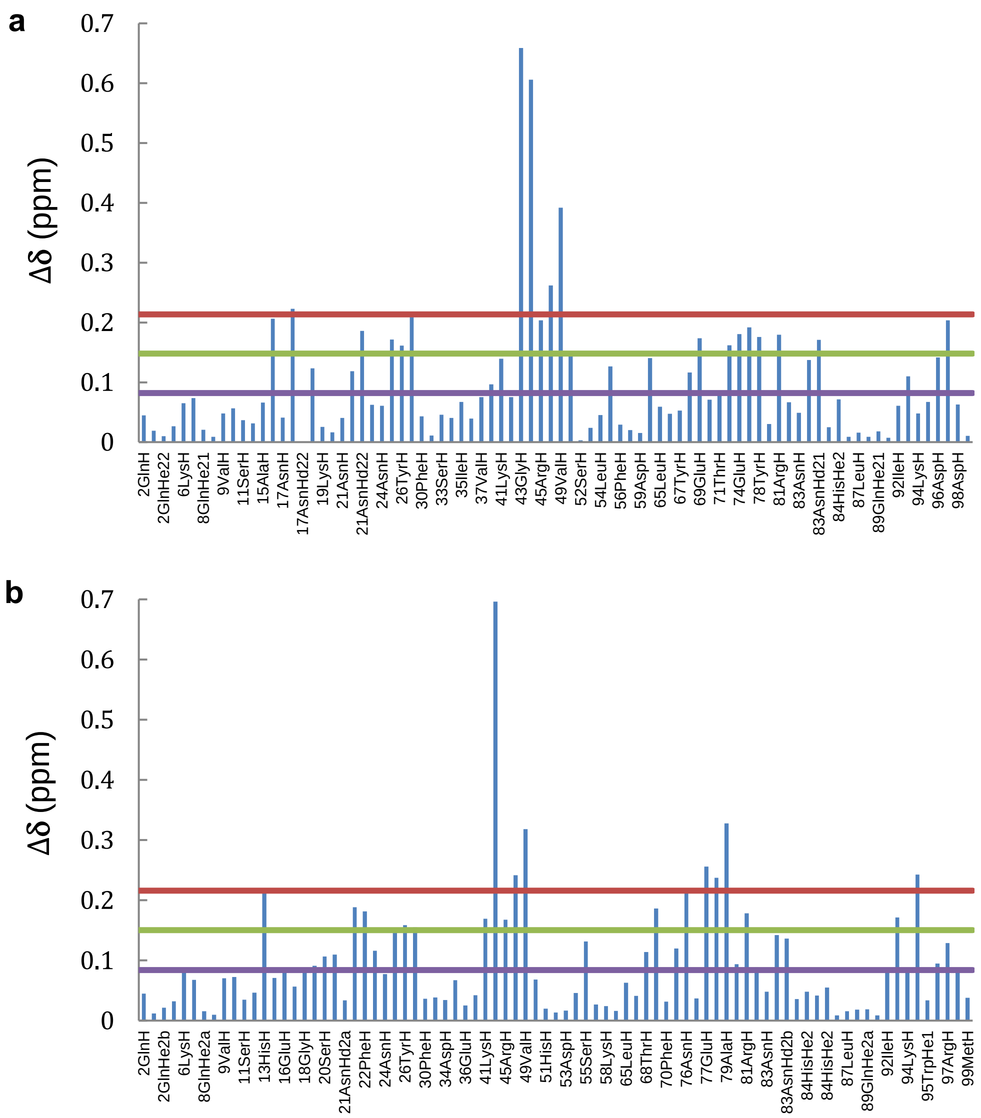

Supplementary Fig. S4. Histograms of ^{15}N-^{1}H HSQC Δδ values as a function of the sequence. The data are relative to wild type (a) and D76N β_2m (b) and were obtained by comparing the spectra of both species in the absence and presence of two-fold molar excess of Nb24. The horizontal lines represent the $\overline{\Delta\delta}$ value, **(**$\overline{\Delta\delta} + \sigma$) and ($\overline{\Delta\delta} + 2\sigma$), where $\overline{\Delta\delta}$ indicates the chemical shift deviation average.

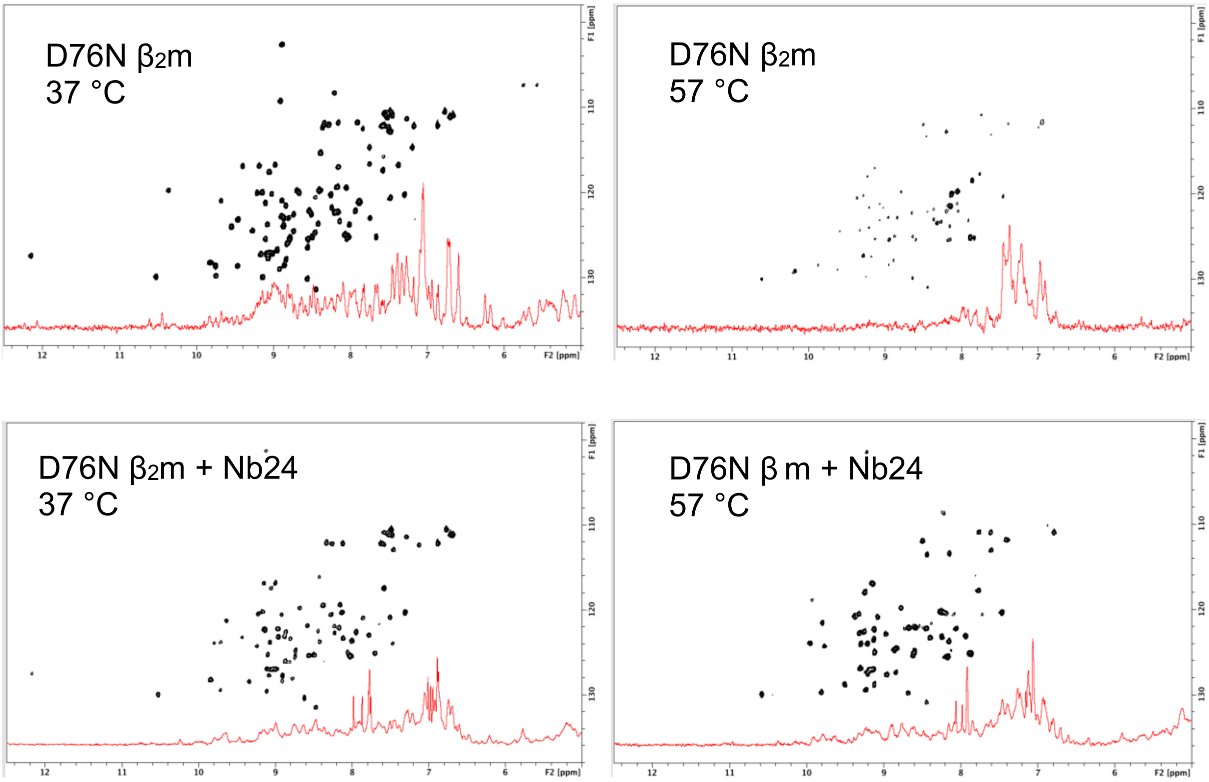

Supplementary Fig. S5. ^{15}N -^{1}H HSQC spectra as a function of the temperature. Overlay of ^{15}N-^{1}H 2D HSQC maps and the corresponding region ^{1}H 1D spectrum for D76N β2m alone or with twofold molar excess of Nb24 at 37 and 57 ºC. In the absence of Nb24 at 57 ºC, the ^{15}N-^{1}H cross-peaks from the folded structure are nearly completely lost, whereas the residual signals in the corresponding region of the 1D spectrum are shrunk into a single unresolved envelope flanking the aromatic proton peaks. This pattern is the signature of the loss of secondary and tertiary structure. On the contrary, in the presence of Nb24, the ^{15}N-^{1}H 2D HSQC map of D76N β2m and the relative ^{1}H 1D spectrum trace still mantain the characteristic resonance spread typical of the NMR pattern of folded proteins.

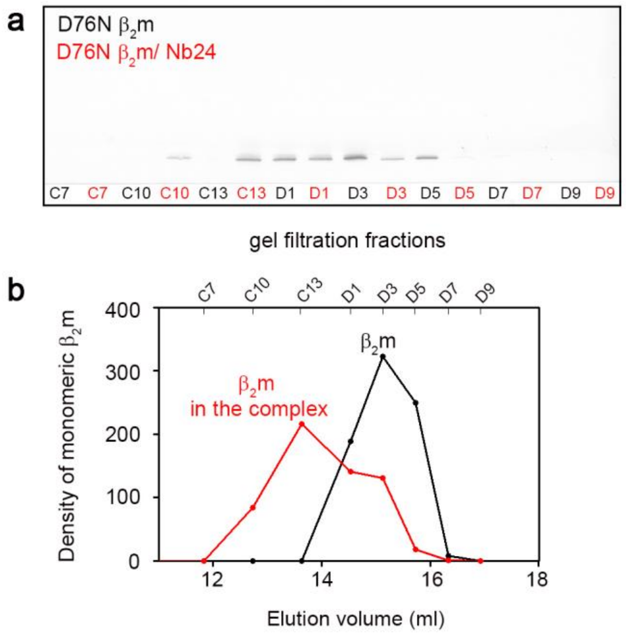

Supplementary Fig. S6. Stability of D76N β$_2$m/ Nb24 in plasma. (**a**) Western blot of fractions from size exclusion chromatography of plasma samples added with D76N β$_2$m with or without Nb24. Identification of β$_2$m in each fraction was carried out as described in the methods section and quantified using Quantity One software. (**b**) Densities of immuno-detected β$_2$m (see panel a) plotted with the corresponding fractions and elution volume indicate the shift in size consistent with the formation of the complex in the presence of Nb24.

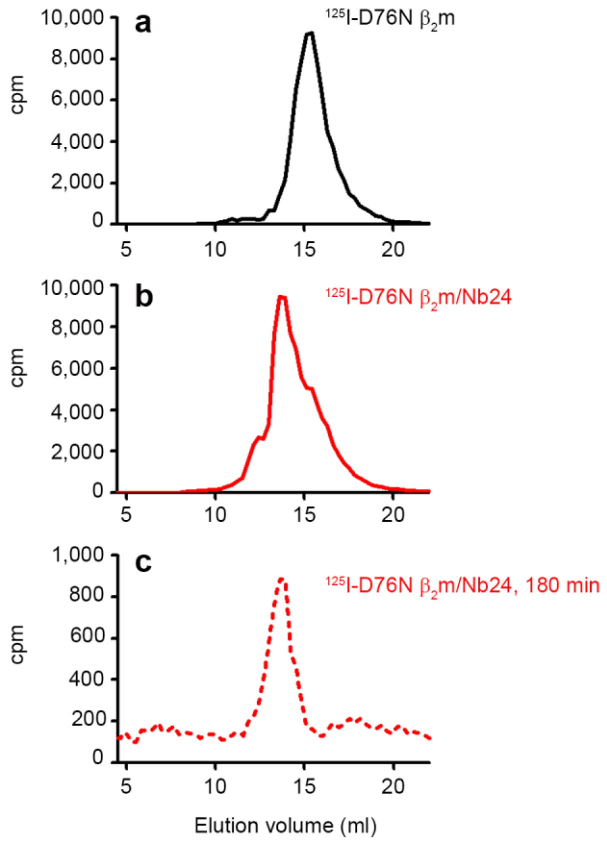

Supplementary Fig. S7. Persistence of D76N β_2m/Nb24 *in vivo*. (**a**) Size exclusion chromatography of ^{125}I-D76N β_2m alone or (**b**) with equimolar quantity of Nb24 in β_2m knock out mouse plasma. (**c**) Size exclusion chromatography of plasma collected at 180 min from β_2m knock out mice receiving ^{125}I-D76N β_2m/Nb24. The species eluted at 180 min (**c**) has the same elution volume of the complex (V_e ~13.8 ml, **b**) compared to the protein alone (V_e~ 15.4 ml, **a**).

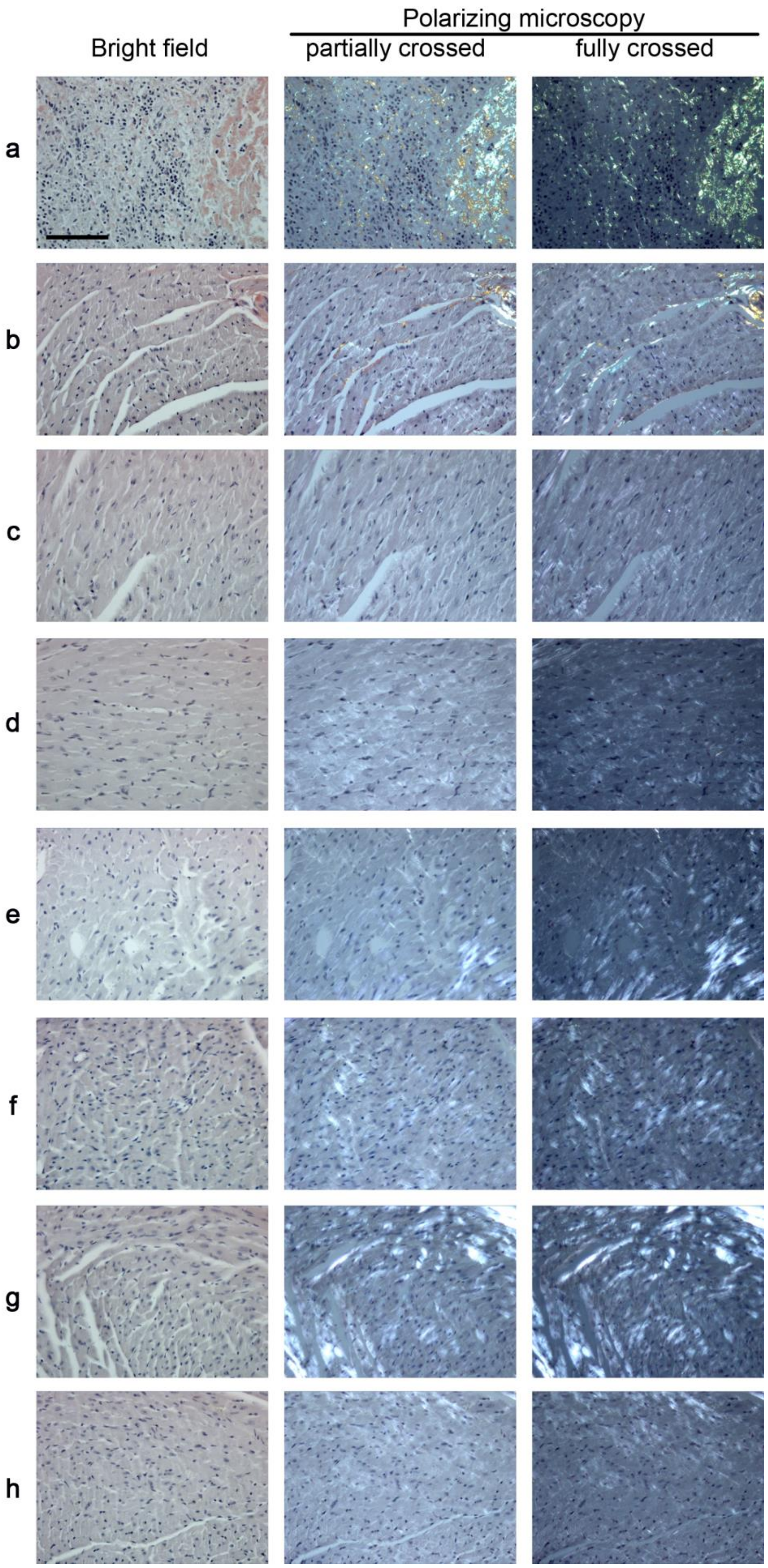
Polarizing microscopy
Bright field
partially crossed
fully crossed
a
b
c
d
e
f
g
h

Supplementary Fig. S8. Histological examination of Congo-red stained sections. (**a**) Clinical specimen as positive control for Congo red staining; (**b**) mouse heart section from the inducible transgenic model of AA amyloidosis[3] in which mice develop minor cardiac amyloid deposits; (**c**-**h**) Congo red stained sections of hearts from β_2m knockout mice treated as follows: (**c**) uninjected control; (**d**) PBS-injected control; (**e**) wild type β_2m injected; (**f**) injected with equimolar mixture of wild type β_2m and nanobody Nb24; (**g**) D76N β_2m injected; (**h**) injected with equimolar mixture of D76N β_2-microglobulin and nanobody Nb24. Sections were stained in parallel with alcoholic alkaline Congo red, and viewed with polarizing microscopy under high intensity illumination[4]. Under these conditions, amyloid is identified the pathognomonic red-green birefringence (**a**) and (**b**). No amyloid deposits were identified in the negative controls or in experimental material (**c-g**). The white birefringence observed in heart sections viewed with polarising is due to the highly ordered arrangement of the myofibrils in cardiomyocytes. Scale bar: 50 µm, applies to all panels. For critical evaluation, on-screen viewing is recommended.

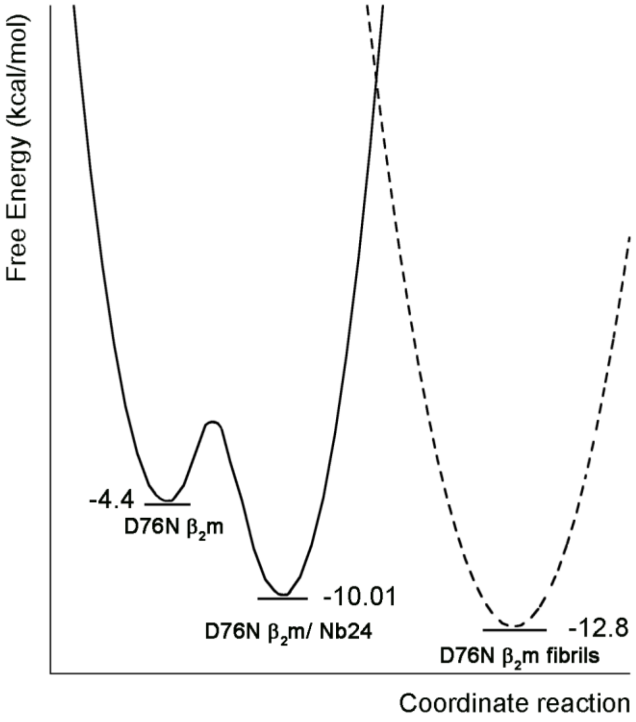

Supplementary Fig. S9. Free energy diagram. Values of free energy (ΔG^0) for equilibrium denaturation of D76N β_2m alone[5] and for D76N β_2m in complex with Nb24 (calculated from the Biacore data) are reported at 37 ºC. The free energy of unfolding of the monomer and of the complex with Nb24 are compared with the free energy of elongation (ΔG^0_{el}) of D76N β_2m fibrils previously measured at 37 ºC[5].

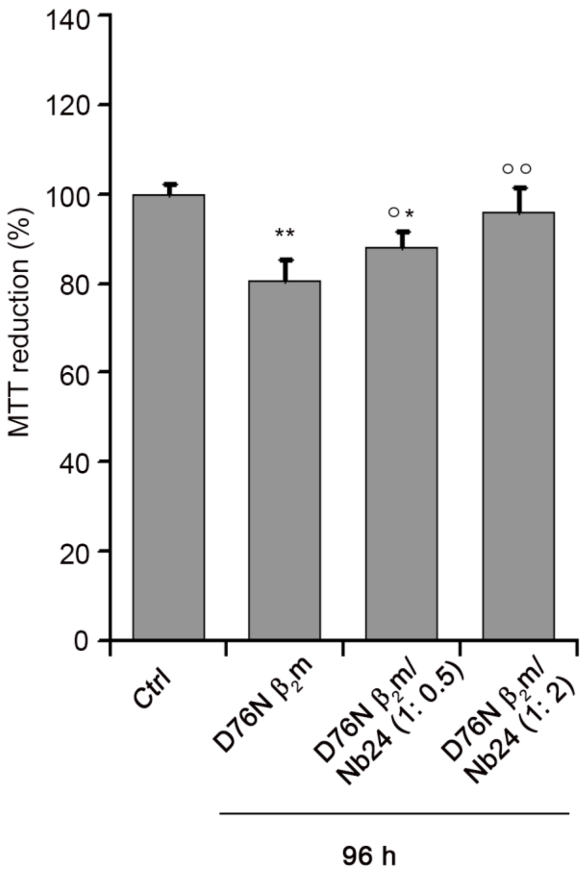

Supplementary Fig. S10. **Nb24 protects against D76N β_2m-induced cytotoxicity.** SH-SY5Y cells were treated with 5.0 μM D76N β_2m after 96 h of aggregation time in the absence or in the presence of Nb24 (1:0.5 and 1:2 β_2m:Nb24 molar ratio). The viability of SH-SY5Y cells was assessed by the MTT reduction assay. Error bars indicate the standard deviation of independent experiments carried out in quadruplicate. *T*-student analysis: *P < 0.05; **P < 0.01; ***P < 0.001 *versus* untreated cells (Ctrl); °P < 0.05; °°P < 0.01 *versus* D76N β_2m aggregates treated cells.

Assessing Nb24 interaction stoichiometry by NMR diffusion measurements. One important issue in the nanobody-antigen interaction is the possible aggregation that may arise following dimer or larger oligomer formation driven either by the nanobody[6] or, possibly, by the target protein. To tackle the problem, all the protein solutions used for NMR were controlled using diffusion ordered spectroscopy (DOSY) measurements. We first collected ^{1}H DOSY data to extract the apparent diffusion coefficients from the isolated Nb24 and β_2m preparations and, subsequently, from the different mixtures of the proteins during the titrations. Supplementary Table S1 and Supplementary Figure S11 summarize the relevant results. In the isotropic motion limit, i.e. assuming spherical species, it can be seen that for isolated Nb24 and β_2m isoforms, as well as for their 2:1 complexes, the determinations are in better agreement with the theoretical values estimated using a protein density calculated according to the reported molecular mass dependence[7]. In fact, the D values calculated from the empirical calibration based on the number of protein residues[8] and recast in terms of molecular mass[9], diverge more from the experimental values. The behavior observed with a specific batch of D76N β_2m that exhibited local resonance doubling, due to a conformational heterogeneity, possibly related to association typically forerunning precipitation or fibrillogenic misfolding, appears sharply different when compared to the pattern of the same variant without any hint of conformational dispersion. The diffusion coefficients and the associated R_h values are similar to the values found with wild type protein that is known to undergo association. The treatment of the data by inverse Laplace transform reduces the number of acceptable D values. Therefore the values extracted for locally polymorph D76N β_2m in the presence of Nb24, despite the suppression of any resonance doubling and therefore conformational heterogeneity, suggest that a fraction of associated protein oligomers still survives after binding to Nb24. Apart from this specific case, however, the data of Supplementary Table S1 confirm that the interaction between the addressed β_2m species and Nb24 occurs essentially with one-to-one stoichiometry.

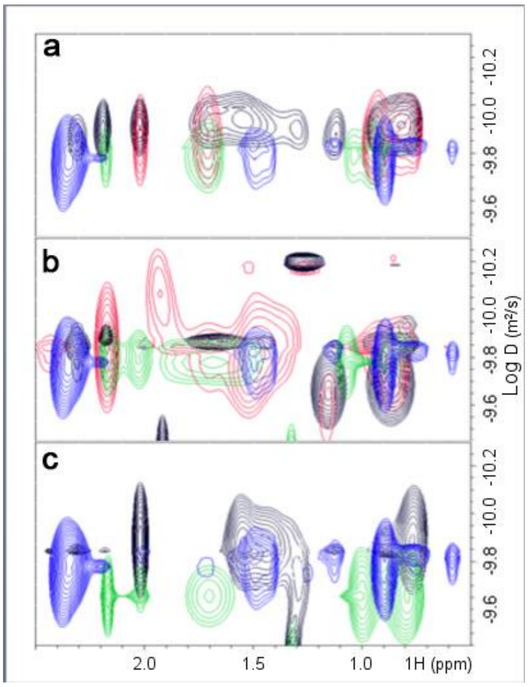

Supplementary Figure S11. **DOSY map comparison of wild type and D76N β_2m.** (**a**) DOSY map overlays of wild type β_2m, (**b**) conformationally heterogeneous D76N β_2m and (**c**) conformationally homogeneous D76N β_2m in the absence and presence of Nb24. In each map the green contours show the isolated protein, the blue contours show isolated Nb24, the red contours (missing in **c**) show the 1:1 mixture and the black contours show 1:2 mixture (1:2.2 in **c**).

Supplementary Table S1. Diffusion coefficients (*D*) and corresponding hydrodynamic radii (R_h), in brackets, determined from DOSY spectra of Nb24, wild type and D76N β_2m preparations at 25 °C[a]. The experimental data are compared to predicted values according to different calibrations. A spherical model is always assumed[b].

Sample	*D* /10^{-10} m^2/s ÷ R_h /10^{-10} m			*Concentration*
	Experimental values	*Model 1*[c]	*Model 2*[d]	
Nb24	1.64, 1.45 [14.7, 16.7]	1.51 [16.0]	1.19 [20.3]	114 μM[e]
D76N β_2m*[f]	1.65, 1.42 [14.6, 17.1]	1.64 [14.7]	1.28 [18.8]	57 μM
D76N β_2m	2.05, 1.91 [11.8, 12.7]	1.64 [14.7]	1.28 [18.8]	54 μM
wild type β_2m	1.69, 1.53 [14.3, 15.8]	1.64 [14.7]	1.28 [18.8]	70 μM
D76N β_2m*-Nb24 1:2	1.42, 1.33, 0.638 [17.0, 18.1, 37.9]	1.24 [20.0]	0.971 [24.9]	57 μM-114 μM
D76N β_2m-Nb24 1:2.2	1.42, 1.27 [17.0, 19.0]	1.24 [20.0]	0.971 [24.9]	75 μM-165 μM
wild type β_2m-Nb24 1:2	1.49, 1.30 [16.2, 18.6]	1.24 [20.0]	0.971 [24.9]	70 μM-140 μM

[a] Other conditions were: solutions in H_2O/D_2O 94/6, 25 mM phosphate, pH* =7.20.

[b] The reported values of *D* were extracted by the inverse Laplace transform routine of Bruker package *Dynamics Center* and may be considered uncertainty ranges of the relevant species diffusion coefficients or occurring species with different diffusion coefficients. The corresponding R_h were always calculated assuming isotropic diffusion and spherical geometry by using equation $D = \frac{kT}{6\pi\eta R_h}$ with *k* Boltzmann constant, *T* absolute temperature, *η* solvent viscosity. The errors on *D* values range between ±1×10^{-12} and ±1×10^{-11} m^2/s.

[c] Model 1: the *D* value was calculated using the spherical approximation equation and by imposing $R_h = \left(\frac{4\pi N_A \rho}{3\, M_w}\right)^{1/3}$ where N_A is the Avogadro number, M_w is the molecular weight (14,971 Da for Nb24, 11,862 Da for wild type β2m and 11,861 Da for D76N β_2m) and *ρ* is the density calculated according to Fischer et al.[7].

[d] Model 2: the *D* value was calculated using the spherical approximation equation and by imposing $R_h = r_0(M_w)^{\nu}$ where M_w is the molecular weight, r_0 = 1.13 and *v* = 0.30[8,9].

[e] The same values were obtained also at a concentration of 232 μM.

[f] The asterisk indicates presence of local conformational heterogeneity.

Supplementary references

1 Karlsson, R. & Falt, A. Experimental design for kinetic analysis of protein-protein interactions with surface plasmon resonance biosensors. *J. Immunol. Methods* **200**, 121-133 (1997).

2 Mosmann, T. Rapid colorimetric assay for cellular growth and survival: application to proliferation and cytotoxicity assays. *J. Immunol. Methods* **65**, 55-63 (1983).

3 Simons, J. P. *et al.* Pathogenetic mechanisms of amyloid A amyloidosis. *Proc. Natl. Acad. Sci. U.S.A.* **110**, 16115-16120, doi:10.1073/pnas.1306621110 (2013).

4 Puchtler, H., Waldrop, F. S. & Meloan, S. N. A review of light, polarization and fluorescence microscopic methods for amyloid. *Appl. Pathol.* **3**, 5-17 (1985).

5 Natalello, A. *et al.* Co-fibrillogenesis of Wild-type and D76N beta2-Microglobulin: the crucial role of fibrillar seeds. *J. Biol. Chem.* **291**, 9678-9689, doi:10.1074/jbc.M116.720573 (2016).

6 Sinacola, J. R. & Robinson, A. S. Rapid refolding and polishing of single-chain antibodies from Escherichia coli inclusion bodies. *Protein Expr. Purif.* **26**, 301-308 (2002).

7 Fischer, H., Polikarpov, I. & Craievich, A. F. Average protein density is a molecular-weight-dependent function. *Protein Sci.* **13**, 2825-2828, doi:10.1110/ps.04688204 (2004).

8 Wilkins, D. K. *et al.* Hydrodynamic radii of native and denatured proteins measured by pulse field gradient NMR techniques. *Biochemistry* **38**, 16424-16431 (1999).

9 Danielsson, J., Jarvet, J., Damberg, P. & Gräslund, A. Translational diffusion measured by PFG-NMR on full length and fragments of the Alzheimer Aβ(1–40) peptide. Determination of hydrodynamic radii of random coil peptides of varying length. *Magn. Reson. Chem.* **40**, S89-S97, doi:10.1002/mrc.1132 (2002).
